# The Emerging Significance of Histone Lysine Demethylases as Prognostic Markers and Therapeutic Targets in Head and Neck Cancers

**DOI:** 10.3390/cells11061023

**Published:** 2022-03-17

**Authors:** Dawid Dorna, Jarosław Paluszczak

**Affiliations:** Department of Pharmaceutical Biochemistry, Poznan University of Medical Sciences, ul. Święcickiego 4, 60-781 Poznan, Poland; dawid.dorna97@gmail.com

**Keywords:** head and neck cancer, epigenetics, histone lysine demethylases, KDM1, KDM4, KDM5, KDM6, KDM inhibitors

## Abstract

Epigenetic aberrations, associated with altered DNA methylation profiles and global changes in the level of histone modifications, are commonly detected in head and neck squamous cell carcinomas (HNSCC). Recently, histone lysine demethylases have been implicated in the pathogenesis of HNSCC and emerged as potential molecular targets. Histone lysine demethylases (KDMs) catalyze the removal of methyl groups from lysine residues in histones. By affecting the methylation of H3K4, H3K9, H3K27, or H3K36, these enzymes take part in transcriptional regulation, which may result in changes in the level of expression of tumor suppressor genes and protooncogenes. KDMs are involved in many biological processes, including cell cycle control, senescence, DNA damage response, and heterochromatin formation. They are also important regulators of pluripotency. The overexpression of most KDMs has been observed in HNSCC, and their inhibition affects cell proliferation, apoptosis, cell motility, invasiveness, and stemness. Of all KDMs, KDM1, KDM4, KDM5, and KDM6 proteins are currently regarded as the most promising prognostic and therapeutic targets in head and neck cancers. The aim of this review is to present up-to-date knowledge on the significance of histone lysine demethylases in head and neck carcinogenesis and to discuss the possibility of using them as prognostic markers and pharmacological targets in patients’ treatment.

## 1. Introduction

Head and neck squamous cell carcinomas (HNSCC) are the sixth most frequent cancer type in humans and were responsible for ~5% of new cancer cases in 2020 [1]. Most HNSCC cases are related to long-term exposure to tobacco and/or alcohol, which are the main risk factors, but a subgroup of (mostly oropharyngeal) tumors are associated with human papillomavirus (HPV) infection and present a better prognosis [2,3]. The molecular heterogeneity of HNSCC tumors stands behind the relatively low effects of currently applied targeted treatments [4]. As with most cancers, therapeutic success is relatively low in a group of patients with advanced stages of the disease. Thus, novel prognostic markers and therapeutic strategies are necessary to improve outcomes. 

The regulation of gene expression is a complex process requiring the precise cooperation of multiple proteins. Epigenetic proteins affect transcription and other DNA-related processes by introducing, reading, and removing covalent groups from DNA and histones, leading to changes in chromatin structure and accessibility for other proteins. Amino acids in histones may undergo reversible acetylation, methylation, phosphorylation, ubiquitylation, etc. All these posttranslational modifications may differentially affect transcription [5,6]. In general, histone lysine acetylation activates transcription. Additionally, active transcription start sites are enriched in H3K4 methylation, while transcription elongation is stimulated by the methylation of H3K36. On the other hand, transcriptional repression is mediated by the methylation of H3K9 and H3K27 sites. By modulating the expression of different sets of genes, changes in epigenetic modifications are implicated in cell plasticity, stemness, differentiation, and other cellular processes. Abnormalities in epigenetic mechanisms are frequently detected in human cancers and can be targeted by epigenetic drugs [7].

Epigenetic aberrations associated with altered DNA methylation profiles and global changes in the level of histone modifications are commonly detected in HNSCC [8,9]. They are related to changes in the profile of expression of epigenetic modifiers in HNSCC [10]. Altered global levels of histone modifications were associated with poor prognosis in oral cancers [11]. Moreover, changes in histone lysine methylation levels in chromatin around transcription start sites were detected in HPV-associated oropharyngeal cancers [12].

Lysine (K)-specific histone demethylases (KDMs) are oxygen-dependent enzymes that can be divided into two groups based on the general mechanism of catalysis and the use of cofactors in the oxidation reaction. KDM1 proteins are FAD dependent, while KDMs belonging to the JumonjiC (JmjC) family contain a Fe^2+^ ion in the active site and require 2-oxoglutarate for their activity. The latter group can be divided into several sub-families (KDM2–KDM8) based on substrate specificity. The biological activity of KDM proteins depends on their enzymatic activity targeted at histone and non-histone proteins but also on catalysis-independent effects [13]. Various KDMs are involved in the transcriptional regulation of several signaling pathways, including the androgen receptor, estrogen receptor, Wnt, or TGF-beta pathways [13].

KDMs are involved in many biological processes such as cell cycle control, senescence, DNA damage response, and heterochromatin formation. They are also important regulators of pluripotency [14]. These roles are important for cell growth, and KDM over-expression is crucial for several types of cancers. By affecting the methylation of H3K4, H3K9, H3K27, H3K36, or other sites, these enzymes play a role in changing the level of expression of tumor suppressor genes and protooncogenes. KDMs may be especially promising as pharmacological targets because they strongly affect the expression of mostly cancer-related genes, which makes them superior to much less specific histone deacetylase inhibitors [15].

Recently, histone lysine demethylases have been implicated in the pathogenesis of HNSCC and have emerged as potential molecular targets. Genomic analyses showed the presence of mutations in KDM4-, KDM5-, and KDM6-encoding genes [16]. Additionally, the expression of several KDMs was elevated in HNSCC, especially in advanced stage cases [17]. Several recent reviews have described the biology and role of particular histone demethylases in human cancers [13,18,19,20,21], but a systematic analysis of their role in HNSCC is lacking. The aim of this review is to present current knowledge on the significance of histone lysine demethylases in head and neck carcinogenesis and to discuss the possibility of using them as prognostic markers and pharmacological targets in patients’ treatment. 

## 2. The Role of KDMs in HNSCC Biology

The first discovery of a histone lysine demethylase (LSD1) was reported in 2004 [18]. Since then, much knowledge has accumulated about the role of KDMs in cancer biology; however, the role of several KDMs (KDM2/3/7/8) is still understudied.

KDM1A (LSD1) is a component of transcriptional co-repressor complexes (e.g., NURD), and it selectively removes the H3K4me1/2 methyl marks and mediates gene repression. The protein is responsible for the regulation of the cell cycle, cell differentiation, pluripotency, or apoptosis, and it shows dysregulation in many types of cancer [18]. There is evidence from in vitro and in vivo preclinical studies that LSD1 plays an important role in the stimulation of cell proliferation and motility and inhibition of apoptosis in HNSCC. A progressive increase in LSD1 content was observed during the transition from hyperplasia through dysplasia to carcinoma in the DMBA- or 4NQO-induced oral cancer mouse model [22]. The overexpression of LSD1 promoted metastatic spread in a mouse model of oral cancer, while the knockdown of LSD1 impaired tumor spread [23]. Another study linked the stimulation of cell proliferation by KDM1A to the induction of E2F1 signaling [24]. Importantly, it was found that LSD1 was enriched in a CD44+/CD133+ subpopulation of CAL27 and HN6 cells, which was considered as a subpopulation of tumor-initiating cells with stem-like properties. This was corroborated by the observation that LSD1 depletion led to reduced tumorsphere formation [22]. 

KDM2 demethylases target H3K36me1/2 and are involved in the regulation of rDNA transcription [13]. KDM2B inhibits cell senescence by modulating the expression of *INK4/Arf* locus genes. Moreover, it promotes cell proliferation and migration as well as self-renewal and stem-like features. Its oncogenic properties were observed in breast, ovarian, gastric, pancreatic, lung, and bladder cancers and were associated with the modulation of AP-1, NF-κB, MYC, and PI3K/Akt signaling pathway [25]. There is some evidence showing the upregulation of KDM2 in HNSCC. KDM2B was found to be overexpressed in HPV+ head and neck carcinomas, and its overexpression was associated with c-MYC copy number gain [26]. KDM2A was also shown to contribute to the hallmarks of head and neck cancers. A study showed that the attenuation of miR-214 by the elevation of the level of circFOXO3 sponge leads to increased expression of KDM2A, which promotes the proliferation, growth, and invasiveness of oral cancer cells in vitro and in vivo [27]. Further research is necessary to elucidate the exact role of KDM2 in HNSCC and the possibility of using it as a therapeutic target.

Histone demethylases from the KDM3 (JMJD1) family, including KDM3A, KDM3B, and KDM3C, can remove the methyl groups from H3K9me2 and lead to the activation of target gene expression [13]. KDM3 proteins are deregulated in multiple cancers [21]. By epigenetically activating Wnt target gene transcription, KDM3 demethylases play an important role in the formation and survival of human colorectal cancer stem cells (CSCs), and KDM3 ablation decreased the chemoresistance of these CSCs [28]. Little is known about the exact role of KDM3s in HNSCC. On the other hand, HNSCCs share some important characteristics with esophageal carcinomas, which allows for some extrapolation. In this regard, it was shown that hypoxia-related induction of the Hif-1α transcription factor stimulated the expression of KDM3A and KDM6B in esophageal cancer cells. This elevation of KDM3A expression was shown to be responsible for the radioresistance of hypoxic cancer cells [29]. Thus, as with KDM2, further research is required to elucidate the significance of KDM3 for HNSCC biology and the effects of its inhibition. 

All the four members of the KDM4 (JMJD2) subfamily (4A, 4B, 4C, and 4D) can catalyze the demethylation of H3K9me2/3. Furthermore, KDM4A–C can also demethylate H3K36me3. H3K9me2/3 is a transcriptional suppressive mark that is associated with the formation of heterochromatin. On the other hand, H3K36me3 is generally believed to inhibit transcription at the transcription start site but to facilitate transcriptional elongation. Thus, KDM4s are considered to be transcriptional activators [20]. Indeed, the over-expression of KDM4A promotes transcription in some types of cancers. KDM4 proteins act as co-activators of some nuclear receptors, which may be crucial for the aberrant growth of cancer cells [30]. KDM4s are overexpressed or deregulated in multiple cancers, including breast, ovarian, lung, prostate, bladder, liver, pancreatic, gastric, and colorectal tumors [20]. KDM4B was shown to cooperate with β-catenin and facilitate β-catenin-driven transcription and enhance gastric cancer metastasis and colon cancer cell proliferation. KDM4C is a hypoxia-inducible factor 1α (HIF-1α) co-activator that enhances the expression of several genes associated with the metabolism of glucose and other HIF-1α related genes [19]. Moreover, the inhibition of KDM4 led to cell differentiation by the stimulation of the expression of E-cadherin and a decrease in the level of Vimentin or Snail in gastric cancer cells [31]. Thus, KDM4 proteins may play crucial roles in regulating metabolic changes acquired by cancer cells and in regulating cell differentiation, adhesion, and migration capacity. KDM4A was shown to promote the proliferation, migration, and invasion of nasopharyngeal cancer cells [32]. Several KDMs (2B, 4A, 4C, 6A, 8) were shown to be associated with the capacity for SCC cancer cell invasion, and the knockdown of KDM4A reduced lymph node metastasis in an orthotopic mouse model of SCC of the floor of the mouth. Mechanistically, the inhibition of KDM4A was able to diminish HGF or EGF-induced cell invasion by attenuating the expression of AP-1 transcription factors and AP-1 target genes (*VEGFA*, *PLAUR*, *IL-8*, *CTGF*) [33].

The inhibition of KDM4A reduced the number of cancer stem cells and inhibited tumorsphere formation in SCC cells [34]. Additionally, the expression of KDM4C was increased in ALDH+ esophageal cancer cells, and its knockdown reduced the percentage of ALDH+ stem-like cells and sphere and colony-forming ability in KYSE150 cells [35].

KDM5 proteins catalyze the demethylation of H3K4me2/3 residues and are responsible for the regulation of the cell cycle and cell differentiation [13]. Much evidence indicates that KDM5 (JARID1) demethylases are potential targets for cancer therapy [19,36]. KDM5A is amplified in many types of cancer, including head and neck carcinomas [37]. KDM5A/B promotes G1/S progression by upregulating cyclin D1 and suppressing p15, p16, p21, and p27. The knockdown of KDM5A resulted in the loss of clonogenic potential, cell cycle arrest at the G1 phase, and increased levels of cyclin-dependent kinase inhibitors p21 and p27 in a variety of cancer cell lines. Elevated expression of KDM5A/B has been detected in a small subpopulation of slowly proliferating, tumor-initiating cells that show intrinsic resistance to a wide variety of cancer therapeutics, including cytotoxic and targeted agents [38]. Furthermore, KDM5s have been shown to promote epithelial-to-mesenchymal transition (EMT), invasion, and metastasis [36]. The expression of KDM5C was elevated in nasopharyngeal carcinomas, and its silencing suppressed cell proliferation and colony formation, induced G1 cell cycle arrest, and significantly induced apoptosis, as well as reduced cell migration and invasion [39]. The stable knockdown of KDM5B suppressed HNSCC cell growth in vitro and in vivo. It induced G1 arrest by downregulating cyclin D2 and upregulating p16. Moreover, KDM5B knockdown stimulated apoptosis by elevating the expression of Bak and reducing the expression of anti-apoptotic Bcl-2 [40]. KDM5B promotes cell migration and invasion by increasing the expression of mesenchymal markers Slug and Vimentin [41]. 

KDM5B promotes cancer stem cell-like properties by elevating the expression of CD113, Oct4A, KLF4, and Nanog. It was hypothesized that KDM5B is responsible for the formation of tumor-initiating cells by promoting the reprogramming and de-differentiation of non-malignant oral cancer cells into stem-like cells. The expression of KDM5B correlated with tumor growth and cell motility. Moreover, it affected the ability to form colonies and tumorspheres [41]. In another study, cells with high expression of KDM5B were identified as cancer stem-like cells showing high expression of CD44 and ALDH1 and increased activity of PI3K. Indeed, the activation of PI3K signaling was found to be crucial for the stimulation of KDM5B activity, which led to the transition of quiescent G0 cells into low-turnover stem-like cells [42].

KDM6A (UTX) and KDM6B (JMJD3) demethylate H3K27me2/3 leading to the transcriptional activation of target genes [13]. Both demethylases are important for developmental processes and cell differentiation. KDM6A is a component of complexes formed by MLL3/4 methyltransferases, which activate gene expression by inducing the demethylation of H3K27me3 and methylation of H3K4 residues [43]. The expression of KDM6B is induced by inflammatory reactions, viral infections, and by oncogenic stimuli. It regulates immune response and cellular senescence [44]. 

HNSCC cells were characterized by mutations and copy number variation of several epigenetic modifiers, including KDM6 proteins [16]. Additionally, the expression of KDM6A and 6B was elevated in primary HNSCC, and KDM6A expression was significantly increased in grade 4 carcinomas [17]. Moreover, HPV-positive cervical and head and neck tumors expressed higher levels of KDM6A, which was required for the HPV-mediated upregulation of *CDKN2A* expression [45]. Moreover, the knockdown of KDM6B led to reduced levels of stemness markers, i.e., the reduced expression of CD44, Bmi1, Nanog, and Oct4, and diminished the proportion of side population cells in esophageal cancer cells [46].

KDM7A and KDM7B (PHF8) act preferentially on mono- and di-methylated repressive marks (H3K9me1/2, H3K27Me2, and H4K20Me1), leading to transcriptional activation. They can also bind H3K4me3, which stimulates their demethylase activity towards H3K27 (KDM7A) or H3K9 (KDM7B). PHF8 has been associated with oncogenic properties in several types of tumors, including liver [47] and breast [48] cancer; however, a paucity of data exists regarding HNSCC. The upregulation of PHF8 was observed in laryngeal and hypopharyngeal carcinomas [49]. Moreover, the knockdown of KDM7B in esophageal cancer cells reduced cell proliferation, migration, and invasion and increased apoptosis [50]. Thus, further studies are necessary to establish the exact role of KDM7 proteins in HNSCC.

KDM8 exerts diverse effects on histone tails, including the demethylation of H3K36me3. It is involved in cell cycle regulation by mediating the expression of p21 and cyclin A1. Several recent reports have shown the prognostic and therapeutic potential of KDM8 in HNSCC. KDM8 (JMJD5) was overexpressed in patients with oral cancers [51]. The knockdown of KDM8 increased the expression of E-cadherin and reduced the expression of mesenchymal markers—N-cadherin, Vimentin, MMP-2/9, Twist, Slug, and α-SMA. Moreover, it led to the activation of both extrinsic (increase in FADD and TRAIL level) and intrinsic (reduction in Bcl-2/Bcl-xL, increase in Bax level) pathways of apoptosis (shown by the elevated level of cleaved caspases-9/8/3). Furthermore, its silencing reduced xenograft tumor growth [51].

Overall, evidence supports the crucial role of KDMs in the regulation of cell proliferation and apoptosis via the transcriptional modulation of the cell cycle and apoptosis modulatory proteins. Moreover, the increased expression of KDMs triggers the epithelial-to-mesenchymal transition and thus stimulates cell migration and invasion, contributing to cancer progression. Importantly, at least some KDMs (LSD1, KDM4A, KDM5B, KDM6B) are among the key modulators of stemness/pluripotency markers in head and neck cancer stem cells, which suggests their potential significance for tumor recurrence.

The regulation of KDM activity in HNSCC is still understudied. However, some evidence shows that the action of KDMs is regulated by several oncogenic signaling pathways which are implicated in HNSCC carcinogenesis: AP-1, c-MYC, EGFR, PI3K, or Wnt/β-catenin.

## 3. Prognostic Significance of KDMs in HNSCC

A certain number of studies have found that the frequently observed upregulation of KDMs has prognostic significance in HNSCC. Most available evidence points to the relevance of the overexpression of LSD1, KDM4, KDM5, or KDM6 for survival or metastasis prediction in OSCC patients.

In comparison to the normal oral mucosa, oral cancers were characterized by the elevated content of LSD1 protein. In addition, LSD1 overexpression was associated with aggressive clinicopathological features, shorter overall survival, and lymph node metastasis in OSCC patients [22]. Additionally, other studies have shown the aberrant overexpression of LSD1 in oral cancers, which was associated with an unfavorable prognosis [52,53]. Moreover, increased LSD1 protein expression was found in malignant oral and laryngeal tumors, and it correlated with tumor stage [23]. 

Some evidence also points to the prognostic importance of KDM4 upregulation in HNSCC. Based on TCGA data analysis, it was found that the expression of all KDM4 isoforms correlated with tumor grade in HNSCC patients, and grade 4 tumors showed the highest increase in KDM4C expression [17]. The level of KDM4A protein was significantly higher in primary SCC than in adjacent epithelium [33]. Moreover, KDM4A was differentially expressed in oral cancer tissues from patients characterized by different survival times, and the increased expression of KDM4A was also associated with the advanced TNM stage, indicating that KDM4A may have significance for the prognosis of OSCC patients [54]. The increased expression of KDM4A was also observed in nasopharyngeal carcinomas, and it correlated with clinical stage, metastasis, and worse prognosis [32]. The level of KDM4A protein was significantly increased in metastatic lymph nodes in comparison to primary human squamous cell carcinomas, which links KDM4A with promoting metastasis in this type of cancer [54]. Indeed, KDM4A content was significantly higher in lymph node metastases [33]. Additionally, the increased nuclear expression of KDM3A was found in OSCC patients and was associated with tumor stage and the risk of lymph node metastasis [55]. 

It has been shown that KDM5B is not expressed in the normal oral epithelium and shows upregulation in HNSCC cell lines and tumors, in which it showed a positive association with Ki-67 labeling [40]. Its overexpression in OSCC patients was associated with reduced survival [41]. Another study also showed that the expression of KDM5B was higher in HNSCC than in adjacent noncancerous tissues. It was also associated with lymph node metastasis and tumor recurrence. Furthermore, patients with high KDM5B expression had shorter disease-free and overall survival times [56]. Moreover, the expression of KDM5C was elevated in nasopharyngeal carcinomas [39]. 

The overexpression of KDM6A was associated with lymph node involvement and was related to shorter disease-free and overall survival in tongue carcinoma patients [57]. Similarly, KDM6B was overexpressed in esophageal squamous cell carcinomas, and it was associated with lymph node metastasis [46,58]. Importantly, the combined overexpression of LSD1 and KDM6B was associated with the worst prognosis in HNSCC patients [59].

Finally, the high expression of PHF8 was associated with clinical stage, shorter overall and disease-free survival, and tumor relapse in laryngeal and hypopharyngeal carcinomas [49]. Additionally, KDM8 high expression was associated with tumor size, lymph node metastasis, and worse survival rate in oral cancer patients [60].

Table 1 presents a summary of the role of KDMs in HNSCC.

## 4. Pharmacological Inhibition of KDMs in HNSCC

The recent development of small molecule inhibitors of KDM proteins allowed the evaluation of the therapeutic potential of their pharmacological inhibition in HNSCC. For some KDMs selective inhibitors are not commercially available; thus, current knowledge pertains mostly to KDM1, KDM4, KDM5, and KDM6 pharmacological inhibition (Table 2).

The chemical (using pargyline or tranylcypromine) or siRNA-mediated inhibition of LSD1 impaired cell proliferation, cell migration, and invasion and also induced apoptosis in CAL27 and HN6 cells [22]. Additionally, melatonin was found to diminish LSD1 expression and activity, which was reflected by increased levels of H3K4 methylation. Both melatonin and pargyline, another LSD1 inhibitor, reduced the proliferation of oral cancer cells [52]. 

Small molecule LSD1 inhibitors reduced xenograft tumor growth [22]. A small-molecule inhibitor of LSD1, GSK-LSD1, reduced tumor size in patient-derived tumor xenografts. It was shown that this compound impaired cell proliferation by attenuating EGFR signaling. This was associated with a reduction in the level of phosphorylated Akt and ERK1/2. GSK-LSD1 also inhibited c-Myc, Wnt/β-catenin, and YAP/TAZ signaling pathways and reduced the expression of *CTGF*, a marker of epithelial–mesenchymal transition (EMT). Moreover, it was shown that GSK-LSD1 promoted p53 expression and induced apoptosis [23]. Thus, the pharmacological inhibition of LSD1 may be a promising anti-cancer strategy, leading to reduced tumor growth and spread by modulating oncogenic signaling pathways responsible for the regulation of cell proliferation, migration, and apoptosis.

Several in vitro studies on the effects of pharmacological inhibition of KDM6 in HNSCC cells have been published. A selective KDM6 inhibitor–GSK-J4, reduced the viability of HNSCC cell lines at low concentrations [17,75]. The treatment of tongue carcinoma cells with GSK-J4 diminished cell proliferation by downregulating cyclin D1. It also reduced the capacity of cells for migration and invasion, and these effects could be attributed to switching cadherin expression by elevating E-cadherin and decreasing N-cadherin [57]. GSK-J4 was also shown to reduce the viability, proliferation, and migration and to induce cell cycle arrest and apoptosis of esophageal cancer cells [46,58].

Interestingly, the combinatorial treatment of HNSCC cells with tranylcypromine (an LSD1 inhibitor) and GSK-J1 (a KDM6 inhibitor) synergistically impaired cell proliferation and induced senescence and apoptosis. These effects were associated with the induction of the expression of Bax, p16, and p21 and the reduction in the level of cyclin D. Moreover, it suppressed tumor growth in the 4NQO-induced and xenograft HNSCC mouse models and led to the appearance of less invasive tumors [59]. 

Some experimental evidence points to the therapeutic potential of targeting KDM4. The selective KDM4 inhibitor ML324 reduced the viability of HNSCC cell lines [17,75]. Furthermore, the inhibition of KDM4A induced DNA replication stress and stalled replication in HNSCC cells [34].

The use of the inhibitor of KDM5B CPI-455 depleted the fraction of CD44+ and ALDH+ oral cancer stem cells and attenuated tumorsphere formation, while it did not affect cell viability or apoptosis. The compound reduced the expression of Oct4 and SOX2 stem cell markers. The pretreatment of cells showing high expression of KDM5B with CPI-455 impaired the formation of xenograft tumors. Additionally, siRNA-mediated silencing of KDM5B significantly induced E-cadherin expression, which showed that the effects of KDM5B on cell motility and invasion are independent of demethylase activity [68]. 

Moreover, plant-derived compounds can modulate KDM activity. In this regard, it was shown that silibinin inhibited KDM8 in oral cancer cells [60].

Importantly, small molecule KDM inhibitors can improve the anti-cancer effects of other treatment strategies. In this regard, LSD1 knockdown sensitized cells to 5-fluorouracil [22], while the inhibition of KDM5B improved radiosensitivity in oral cancer cells [41]. The chemical inhibition of KDMs with a non-selective inhibitor IOX-1 (which targets mainly KDM3) or KDM3 knockdown in esophageal cancer cells also increased radiosensitivity and decreased tumor size in vivo [29]. Since radiotherapy is frequently applied in the treatment of HNSCC patients, it is intriguing whether KDM3 or KDM5 inhibition could be used clinically for radiosensitization.

Moreover, ML324 and GSK-J4 synergistically increased the pro-apoptotic activity of EGFR or PI3K signaling inhibitors—erlotinib or HS-173, respectively—in HNSCC cells [17]. Additionally, the elevated expression of KDM5A was observed in erlotinib-tolerant persister head and neck cancer cells. The silencing of KDM5A increased the expression of *CDH1* and reduced the expression of *Vimentin*, suggesting that KDM5A regulates the expression of mesenchymal markers in persister cancer cells [67]. It has to be evaluated clinically whether KDM5 inhibitors could prevent relapse by eliminating resistant, therapy-persister, and stem-like cells that are responsible for recurrence. In addition, the inhibition of KDM4A enhanced the therapeutic activity of PD1 blockade immunotherapy. Such combinatorial treatment inhibited SCC growth and metastasis in the 4-NQO-induced SCC mouse model. This activity was associated with the activation of anti-tumor immunity via the recruitment, intratumoral infiltration, and activation of CD8+ T cells. Moreover, such combinatorial treatment led to the elimination of cancer stem cells [34]. Thus, joint therapy with KDM4-6 inhibitors may potentially improve the efficacy of EGFR and/or PD1 inhibitors in refractory HNSCC patients. 

Most evidence of the anti-cancer effects of the inhibition of LSD1 and KDM4-6 comes from in vitro studies, but there is also some confirmation of such activity coming from preclinical in vivo models. While more research is necessary to critically evaluate the therapeutic potential of KDM inhibitors in HNSCC, the available evidence suggests that targeting KDMs can be regarded as one of the novel promising strategies for HNSCC chemotherapy.

## 5. Conclusions

There is strong evidence that the dysregulation of KDM1, KDM4, KDM5, and KDM6 proteins is frequently observed and plays a significant role in head and neck carcinogenesis. This evidence comes from the clinical observations of the prognostic value of the overexpression of KDM demethylases and preclinical experiments, both in vitro and in vivo, which point to the high therapeutic potential of KDM inhibitors. On the other hand, much less is known about the role of other histone lysine demethylases in HNSCC, with only single reports in the case of KDM3 and KDM7. However, it can be hypothesized that these proteins have rather oncogenic properties in HNSCC. Thus, there is an important question regarding the selection of pharmacological targets that would have the greatest therapeutic potential. Is targeting individual demethylases better than using combinations of inhibitors or non-selective pan-KDM inhibitors? If targeting individual demethylases would seem a better strategy, then which of the KDMs should we target? In this regard, can HNSCC patients be stratified by sensitivity to different KDM inhibitors? These questions would have to be answered in the future to warrant the clinical usefulness of KDM inhibitors. Based on current evidence, we can speculate that targeting two or several KDMs may be beneficial, as shown in the case of combinations of tranylcypromine (an LSD1 inhibitor) and GSK-J1 (a KDM6 inhibitor) [59]. Moreover, targeting several KDMs may be a more universal strategy due to the differences in response to individual KDM inhibitors, e.g., FaDu cells were more susceptible to GSK-J4 action in comparison to CAL27 cells, which showed a stronger response to ML324 [17]. In order to more systematically answer these questions, future research should focus on dissecting the exact role played by individual KDMs in transcriptional control in HNSCC cells by analyzing their interaction partners (e.g., transcription factors) and sets of upregulated/downregulated cancer-related genes under different conditions (e.g., normoxia vs. hypoxia). Moreover, evidence from patient-derived organoids is also needed.

However, more questions need to be addressed before KDM inhibitors enter clinical trials. Since KDMs are oxygen-dependent enzymes, low levels of oxygen may affect their catalytic activity. The effects of hypoxia on KDM action need to be further elucidated. Some studies suggest decreased activity in hypoxic environments [36,76,77,78]. On the other hand, hypoxia-induced HIF transcription factors may cooperate with KDMs in transcriptional regulation [14]. Thus, the effect of KDM inhibitors on cells in hypoxic compartments in head and neck tumors has to be further assessed. 

**Figure 1:**
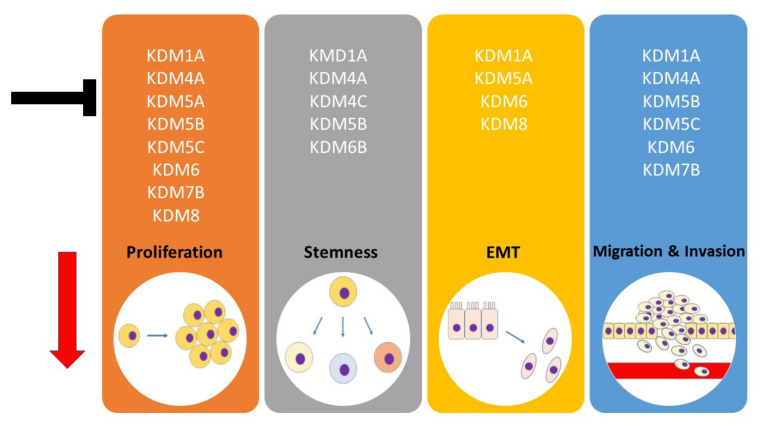
The effects of KDM inhibition or knockdown on critical HNSCC hallmark features.

Altogether, KDM inhibitors provide a new therapeutic opportunity in the treatment of HNSCC patients because they have the potential to target crucial hallmark features which are associated with cancer progression: cell proliferation, senescence, cell plasticity, stem-like characteristics, and also epithelial–mesenchymal transition and cell motility (Figure 1). Their exact utility as chemotherapeutics has to be assessed in future preclinical and clinical studies. 

## Figures and Tables

**Table 1:** The role of histone lysine demethylases in head and neck cancers.

Histone Lysine Demethylase	Target Site	Tumor Localization	Significance	Reference
KDM1A (LSD1)	H3K4me1/2 H3K9me1/2	Oral cavity	Involved in the cell cycle and proliferation by modulating E2F signaling. Overexpression is associated with poor clinical outcomes.	[24]
		Tongue	Aberrantly overexpressed in a significant fraction of tongue SCC. High expression promotes cancer cell growth, proliferation, and metastasis as well as correlates with tumor size, pathological grade, and poor prognosis. Involved in the regulation of the microenvironment and EMT.	[23,52,53]
		Esophagus	Expression is higher in esophageal cancer tissues than in normal tissues and correlates with lymphovascular invasion, high tumor stage, and poor prognosis. Involved in cell growth and proliferation. Role in migration, invasion, and EMT. Contributes to Warburg’s effect by promoting glucose uptake and a metabolic shift toward glycolysis.	[61,62,63]
KDM2A (JHDM1A/ FBXL11)	H3K4me3 H3K36me1/2	Tongue	Involved in cancer cell proliferation and tumor progression.	[27]
KDM2B (JHDM1B/ FBXL10)	H3K36me1/2 H3K4me3	Larynx	Overexpressed in a subset of HPV-positive laryngeal squamous cell carcinomas. Associated with c-MYC copy number gain.	[26]
KDM3A (JHDM2A/ JMJD1A/ TSGA)	H3K9me1/2	Oral cavity	Nuclear expression is associated with a 10-fold increase in lymph node metastasis risk.	[55]
		Hypopharynx/larynx	Hypoxia-related regulator of carcinogenesis.	[64]
		Esophagus	Role in hypoxia-related radioresistance and cancer progression.	[29]
KDM4A (JMDM3A/ JMJD2A)	H3K4me3 H3K9me2/3 H3K36me2/3	Oral cavity/ larynx	Involved in immune evasion and invasive growth. Targeting KDM4A enhances anti-PD-1 therapy and eliminates cancer stem cells.	[34]
		Nasopharynx	Overexpression correlates positively with tumor stage, metastasis, and clinical stage. Role in promoting cancer cell proliferation, migration, invasion, and Warburg effect.	[32]
		Larynx/ hypopharynx/oral cavity	Frequently overexpressed compared to normal epithelia. The high abundance of this protein is associated with metastasis, and its depletion reduces the invasive potential of SCC cells. Involved in the regulation of the JUN and FOSL1 expression.	[33]
KDM4C (JMDM3C/ JMJD2C/ GASC1)	H3K4me3 H3K9me1/2/3 H3K36me2/3	Esophagus	Gene frequently amplified in esophageal SCC. Its high expression is associated with poor survival. Role in stemness promotion via NOTCH1 promoter demethylation.	[35,65]
KDM5A (JARID1A/RBP2)	H3K4me2/3 H3K9me1/2	Head and neck	One of the 8 genes amplified in both cell lines and tumors in genomic analysis, involving 39 HNSCC cell lines and 106 HNSCC tumors.	[66]
		Tongue/larynx	Role in the regulation of the EMT and ferroptosis susceptibility.	[67]
KDM5B (JARID1B/ PLU-1)	H3K4me2/3 H3K36me3	Head and neck	Frequently overexpressed in different types of HNSCC. Upregulation is associated with progression parameters, including lymph node metastasis and recurrence. Knockdown results in cell cycle arrest and apoptosis by suppressing Bcl-2 family members.	[40,56]
		Tongue/ Oral cavity	Frequently upregulated with a role in migration, invasion, stemness, EMT, and radioresistance. Its catalytic activity is not required to sustain parts of its prooncogenic functions, like repressing E-cadherin and promoting invasion.	[41,42,68]
		Hypopharynx	Possible role as a tumor suppressor by promoting differentiation and inhibiting proliferation.	[69]
		Esophagus	Downregulation by miR-194 results in inhibition of cancer cells proliferation and invasion along with intensified apoptosis.	[70]
KDM5C (JARID1C/ SMCX)	H3K4me2/3 H3K9me3 H3K27me3	Esophagus	Its inhibition entails upregulation of apoptosis-related genes and reduces cell proliferation.	[71]
KDM6A (UTX)	H3K27me2/3	Head and neck	Its expression is altered in about a third of HNSCC cases. Frequently overexpressed in HPV-positive tumors. Activity of this histone demethylase is required to maintain p16 expression, which is necessary for HPV E7 expressing cancer cells, despite the tumor-suppressing role of p16 in most cancers.	[16,45]
		Esophagus	High expression is associated with a better prognosis, and downregulation increases cell growth and reduces E-cadherin expression. Role in hypoxia-related radioresistance.	[29,72]
		Tongue	Overexpression is associated with a poor prognosis in patients after surgical resection. Involved in the regulation of the cell cycle, EMT, and invasion.	[57]
KDM6B (JMJD3)	H3K9me3 H3K27me2/3	Tongue/hypopharynx	Simultaneous inhibition of LSD1 and JMJD3 impairs cell proliferation and induces apoptosis and senescence.	[59]
		Oral cavity/ tongue/ hypopharynx	Role as a tumor suppressor. Repression by Notch-effector CSL promotes proliferation and tumorigenesis.	[73]
		Esophagus	Overexpression is associated with poor prognosis. Upregulation is especially pronounced in patients with lymph node metastasis. Important role in the regulation of many signaling pathways involved in cancer cells proliferation, stemness, invasion, and susceptibility to therapy.	[46,58,74]
KDM7B (PHF8)	H3K4me3 H3K9me1/2 H4K20me1 H3K27me2	Larynx/ hypopharynx	High expression is associated with shorter survival and disease-free survival. Overexpression correlates positively with T classification, clinical stage, and tumor relapse.	[49]
		Esophagus	Knockdown results in inhibition of cancer cells proliferation, an increase in apoptosis, a reduction of colony formation, and a drop in the number of migratory and invasive cells.	[50]
KDM8 (JMJD5)	H3K4me3 H3K9me3 H3K36me2	Tongue/ Oral cavity/ Larynx	Overexpressed in comparison to normal oral mucosa. Suppression entails reduced cancer cell migration and invasion, at least in part through its involvement in the regulation of EMT. Inhibition promotes apoptosis by regulating the activation of caspases and p53.	[51]
		Tongue	Frequently overexpressed, leading to increased proliferation of cancer cells.	[60]

Abbreviations: EMT—epithelial–mesenchymal transition; HPV—human papilloma virus.

**Table 2:** The effects of KDMs pharmacological targeting in HNSCC models.

KDM Inhibitor	Target	Experimental Model	Effects	Reference
Tranylcypromine	LSD1	In vitro: HN6 and CAL27 cell lines In vivo: DMBA- and 4NQO-induced OSCC and xenograft animal models	Impaired cell proliferation, migration, invasion, as well as induced apoptosis and chemosensitivity. Reduced xenograft tumor growth.	[22]
Pargyline	LSD1	In vitro: HN6 and CAL27 cell lines In vivo: DMBA- and 4NQO-induced OSCC and xenograft animal model	Impaired cell proliferation, migration, invasion, as well as induced apoptosis and chemosensitivity. Reduced xenograft tumor growth.	[22]
		In vitro: SAS, SCC25, SCC4, and OEC-M1 cell lines	Reduced cell proliferation and viability.	[52]
GSK-LSD1	LSD1	In vitro: HSC-3 and CAL-27 cell lines In vivo: patient-derived tumor xenografts	Impaired cell proliferation by attenuating EGFR, c-Myc, Wnt/β-catenin, and YAP/TAZ signaling pathways. Reduced expression of EMT-related genes. Promoted p53 expression and induced apoptosis. Reduced tumor size in patient-derived tumor xenografts.	[23]
Melatonin	LSD1	In vitro: SAS, SCC25, SCC4, and OEC-M1 cell lines In vivo: xenograft animal models	Impaired cell proliferation and induced cell cycle arrest in the G0/G1 phase. Reduced xenograft tumor growth.	[52]
IOX-1	KDM3 (dominant target), KDM4, KDM6	In vitro: Kyse-30, Kyse-410, and OE21 cell lines In vitro/in vivo: CAM assay	Increased radiosensitivity. Decreased tumor size in vivo.	[29]
ML324	KDM4	In vitro: CAL27 and FaDu cell lines	Reduced cell viability and increased activity of EGFR and PI3K signaling inhibitors (erlotinib, HS-173).	[17,75]
CPI-455	KDM5B	In vitro: SCC9, OCTT2, CAL33, and VU147T cell lines In vivo: xenograft animal models	Reduced expression of stemness-related genes and attenuated tumorsphere formation without effects on cell viability or apoptosis. Impaired formation of xenograft tumors.	[68]
Combination of GSK-J1 and Tranylcypromine	KDM6B LSD1	In vitro: CAL27, FaDu, and HN6 cell lines In vivo: 4NQO-induced HNSCC and xenograft animal models	Impaired cell proliferation and induced senescence and apoptosis. The effects were linked to increased expression of Bax, p16, and p21, as well as a decrease in cyclin D levels. Suppressed tumor growth and the appearance of less invasive tumors.	[59]
GSK-J4	KDM6	In vitro: CAL27 and FaDu cell lines	Reduced cell viability and increased activity of EGFR and PI3K signaling inhibitors (erlotinib, HS-173).	[17,75]
		In vitro: CAL27 and SAS cell lines	Diminished cell proliferation by downregulating cyclin D1. Reduced cell migration and invasion capacity, which can be linked to elevated E-cadherin and decreased N-cadherin levels.	[57]
		In vitro: K510 and K30 cell lines In vivo: xenograft animal models	Suppressed cell growth and migration. Reduced xenograft tumor growth.	[46]
		In vitro: Kyse-150 cell line	Reduced cell viability, proliferation, migration, and invasion as well as induced cell cycle arrest and apoptosis.	[58]
Silibinin	KDM8	In vitro: SAS, SCC25, and HSC3 cell lines In vivo: xenograft animal models	Suppressed cell proliferation and reduced xenograft tumor growth at least partly through downregulation of KDM8.	[60]
